# Serum IgE Induced Airway Smooth Muscle Cell Remodeling Is Independent of Allergens and Is Prevented by Omalizumab

**DOI:** 10.1371/journal.pone.0136549

**Published:** 2015-09-02

**Authors:** Michael Roth, Feng Zhao, Jun Zhong, Didier Lardinois, Michael Tamm

**Affiliations:** 1 Pulmonary Cell Research, Department Biomedicine, University Basel, Basel, Switzerland; 2 Department Internal Medicine, Pneumology, University Hospital Basel, Basel, Switzerland; 3 Department of Respiratory Diseases, Xijing Hospital, 4th Military Medical University, Xi’an, People’s Republic of China; 4 Department Thoracic Surgery, University Hospital Basel, Basel, Switzerland; French National Centre for Scientific Research, FRANCE

## Abstract

**Background:**

Airway wall remodeling in allergic asthma is reduced after treatment with humanized anti-IgE-antibodies. We reported earlier that purified IgE, without the presence of allergens, is sufficient to induce airway wall remodeling due to airway smooth muscle cell (ASMC) activity deposing extracellular matrix.

**Objective:**

We postulate that IgE contained in serum of allergic asthma patients, in the absence of allergens, stimulates ASMC remodeling activities and can be prevented by anti-IgE antibodies.

**Methods:**

Isolated human ASMC were exposed to serum obtained from: (i) healthy controls, or patients with (ii) allergic asthma, (iii) non-allergic asthma, and (iv) atopic non-asthma patients. Proliferation and the deposition of collagens and fibronectin were determined after 3 and 5 days.

**Results:**

Serum from patients with allergies significantly stimulated: (i) ASMC proliferation, (ii) deposition of collagen type-I (48 hours) and (iii) of fibronectin (24 hours). One hour pre-incubation with Omalizumab prevented these three effects of allergic serum, but had no significant effect on serum from healthy donors or non-allergic asthma patients. Interestingly, the addition of allergens did not further increase any of the IgE effects.

**Conclusion and Clinical Relevance:**

Our data provides experimental evidence that the beneficial effect of Omalizumab on airway wall remodeling and improved lung function may be due to its direct action on IgE bound ASMC.

## Introduction

Allergies cause approximately 60% of all asthma cases and correlate with increased circulating IgE levels, which contribute to chronic inflammation [1]. Beside inflammation airway wall remodeling is a leading pathology in asthma and among other factors it is induced by IgE [2, 3]. To counteract the pathologic effects of IgE in atopic asthma humanized anti-IgE antibodies such as Omalizumab have been introduced as a therapeutic concept, and they have been clinically proven to achieve additional beneficial effects on airway wall remodeling compared to standard therapy by inhaled glucocorticoids and long acting β_2_-agonists [3–5]. Neither the mechanisms through which IgE stimulates airway wall remodeling nor those by which anti-IgE antibodies prevent this pathology is fully characterized [3].

The application of neutralizing anti-IgE antibodies in atopic asthma was accepted as a therapeutic concept first in Australia in 2002 [6]. However, only in 2014 it was discovered that this concept naturally occurs in some asthma patients who produce their own anti-IgE antibodies [7]. The study indicated that the presence of natural anti-IgE antibodies accounts for reduced basophil activity and thus may help to reduce airway inflammation [7]. How the production of these anti-IgE antibodies is induced and if they occur in other allergy diseases needs further investigation. Importantly, this observation supports the concept of therapeutic use of humanized anti-IgE antibodies in allergic asthma and other allergic diseases.

IgE has been shown to contribute to airway wall remodeling and there is no doubt that ASMC express and respond to the high and low affinity IgE receptors, thus a direct effect of IgE on tissue forming cells has to be considered [8–10]. It has been demonstrated that IgE up-regulated proliferation of ASMC particularly in asthma patients and that this effect can be reduced by anti-IgE antibodies such as Omalizumab [8, 9]. Furthermore, we provided evidence that at least *in vitro* IgE-induced deposition of collagen type-I, -III and fibronectin deposition was inhibited by Omalizumab [9]. Our *in vitro* data was supported by a clinical study showing that addition of Omalizumab to conventional asthma therapy over a period of 16 weeks resulted in a significant reduced thickness of the airway wall [11]. A second study reported that Omalizumab therapy over one year reduced the thickness of the reticular basement membrane as well as eosinophil infiltration in asthma patients [12].

In airway wall remodeling two resident sub-epithelial cell types, fibroblasts and airway smooth muscle cells (ASMC), play a crucial role in asthma [13, 14]. Airway wall remodelling is the result of several independent pathologic events in the airway wall including: (i) increased proliferation of mesenchymal cells (ASMC, fibroblasts), (ii) modified differentiation of mesenchymal cells, (iii) *de novo* synthesis, and (iv) deposition of pro-inflammatory extracellular matrix components such as collagen type-I and fibronectin. Primate asthma models and studies in childhood asthma indicated that airway remodeling precedes inflammation upon inhalation of allergens and acts through the activation of ASMC and fibroblasts [15, 16]. *In vitro* serum obtained from patients with severe allergic asthma induced changes of the extracellular matrix composition as well as it stimulated cell proliferation, however, none of these studies provided direct prove of the role of IgE in these cellular pathologies [17–19].

In this study we investigated if the effect of serum IgE obtained from atopic patients (non-asthma) and of patients with atopic asthma has a different effect on airway remodeling parameters including proliferation, cytokine secretion and extracellular matrix deposition.

## Material and Methods

### Patient cohort, collection of primary cells and peripheral blood sample

Serum was collected from: (i) ten asthmatics with known allergies, (ii) ten non-allergic asthmatics, (iii) ten atopics without asthma; and (iv) ten non-diseased blood donors. Ten milliliters of peripheral blood were collected and serum was isolated by centrifugation (30 min., 800 x g, 4°C).

All patients gave written informed consent and the study was accepted by the local ethical committee (EKBB) under the registration number EK:05/06.

The demographic data of the patients groups is provided in “Table 1”.

**Table 1:** Epidemiologic data of serum donors. Values represent mean ± S.D.

Diagnosis	Gender	Age	IgE (IU/ml)	IL-6 (pg/ml)	Eotaxin (pg/ml)	Known allergies
Healthy	Male	33	11	12	0	None
Healthy	Male	48	0	0	0	None
Healthy	Female	31	8	0	0	None
Healthy	Male	55	0	18	0	None
Healthy	Female	28	0	0	0	None
Healthy	Female	45	0	0	0	None
Healthy	Male	53	0	15	0	None
Healthy	Male	42	0	21	0	None
Healthy	Female	33	0	0	0	None
Healthy	Male	36	0	0	0	None
**Mean Healthy**	6m/4f	**40.4±8.2**	**1.9±3.04**	**6.6±7.12**	**0**	
Atopic	Male	44	62	1223	638	Pollen
Atopic	Female	32	112	547	146	Pollen
Atopic	Male	29	172	638	392	Cat
Atopic	Female	33	88	1123	23	Pollen
Atopic	Male	54	92	537	175	Pollen
Atopic	Male	49	95	128	273	Pollen
Atopic	Male	52	162	873	263	Pollen
Atopic	Male	28	65	126	476	Fish
Atopic	Female	31	109	1524	35	Pollen
Atopic	Male	29	85	337	147	Pollen
**Mean Atopic**	7m/3f	**38.1 ± 9.3**	**104.2 ± 27.7**	**705 ± 384**	**257 ± 152**	
asthma	Male	42	0	1364	286	None
asthma	Male	65	0	534	326	None
asthma	Male	28	0	1665	532	None
asthma	Female	57	166	34	0	None
asthma	Female	77	0	69	589	None
asthma (+COPD)	Male	64	45	527	115	None (Smoker)
asthma	Male	63	0	1125	156	None
asthma	Female	61	16	3318	223	None
asthma	Female	28	0	1427	476	None
asthma	Male	46	0	0	0	None
**Mean Non-atopic**	6m/4f	**53.1±13.7**	**22.7 ±33.1**	**1006 ±774**	**270 ± 172**	
atopic asthma	Female	28	365	1123	527	Dust, pollen
atopic asthma	Male	70	271	656	53	Multiple Allergies
atopic asthma	Female	44	12	432	140	Multiple Allergies
atopic asthma	Female	57	0	1324	34	Pollen
Atopic asthma	Male	42	606	1347	476	Cat, Dog, Pollen
atopic asthma	Male	64	721	1534	164	Cat
atopic asthma	Female	64	242	657	122	Multiple Allergies
atopic asthma	Male	31	182	328	362	Dust, Cat
atopic asthma	Male	51	0	273	185	Multiple Allergies
atopicasthma	Female	78	105	857	284	Multiple Allergies
**Mean atopic asthma**	5m/5f	**52.9±13.7**	**250.4±199.3**	**853 ± 384**	**234 ± 142**	

### Airway smooth muscle cells

Primary, human ASMC were isolated from: (i) five asthmatic, (ii) five non-asthmatic donors. The demographic data of the tissue donors is presented in “Table 2”. All Asthma patients were selected by a specialist according to standard guidelines and IgE levels were determined by routine laboratory assays (abcam, cat# ab108650). Human ASMC were isolated from bronchial biopsies as described earlier [20, 21]. ASMC were propagated in a medium composed of Dulbecco modified eagle medium (DMEM) supplemented with 20 mM HEPES, 8 mM L-glutamine, 1x vitamin mix and 5% fetal calf serum (Gibco BRL, Zug, Switzerland). For serum starvation, fetal calf serum was reduced to 0.1% over 24 hours.

**Table 2:** Clinical characteristic of cell donors for ASMC and fibroblasts.

Diagnosis	Gender	Age	Allergies
Asthma	Male	28	None
Asthma	Male	42	None
Asthma	Female	28	None
Atopic-asthma	Male	71	Multiple Allergies
Asthma	Male	61	None
Atopic asthma	Female	44	Multiple Allergies
**Mean**		**45.6±13.5**	
COPD	Male	68	Not-known
Cancer	Male	76	Not-known
Cancer	Male	50	Not-known
Cancer	Male	60	Not-known
COPD	Male	68	Not-known
Unknown etiology	Male	39	Not-known
**Mean**		**60.2±10.5**	

### Cell treatment

Omalizumab was used at increasing concentrations (0, 0.1, 1.0, 10.0 μg/ml), dissolved in cell culture medium and mixed with serum samples accordingly. In order to block IgE in the serum samples, the Omalizumab treated serum was pre-incubated for 30 and 60 minutes prior to cell stimulation.

For extracellular matrix deposition confluent tissue forming cells were treated for up to 5 days with either: (i) serum from healthy controls; (ii) IgE rich serum of patients with allergies other than asthma; (iii) IgE rich serum of asthma patients with allergic asthma and (iv) serum from asthma patients without known allergies. In some experiments a mixture of asthma relevant allergens (house dust mite, cat, dog allergens: 1 μg/ml each) was added together with the serum.

ASMC proliferation was determined by manual cell count using an improved Neubauer chamber slide. Sub-confluent (80%) cells in 6-well plate were incubated over 5 days with patient’s serum and/or Omalizumab (0.1, 0.5, 1.0 μM) before being trypsinized, re-suspended in 1 ml phosphate buffered saline (PBS) and counted [21].

Omalizumab was dissolved in phosphate buffered saline (PBS) in a stock solution of 10 μg/ml and dilutions were performed in cell culture medium. When assessing the effect of Omalizumab on serum IgE the compound was pre-incubated for 30 minutes with the corresponding serum sample diluted with cell culture medium to the final concentration (0, 0.1, 1.0, 10.0 μg/ml) before being applied to the cells.

### Collagen and fibronectin deposition

This was determined by a house developed ELISA specific for collagen type-I, collagen type-III and fibronectin [22]. Cells were grown to confluence plus 3 days before being stimulated with serum in either the presence or absence of Omalizumab (0.1, 0.5, 1.0 μM) for 48 hours. In brief, cells were washed (3 x 5 min) with PBS containing 2% bovine serum albumin and fixed in 2% para-formaldehyde. Fixed cells were incubated with one of the primary antibodies for overnight (4°C) and then washed 3 x with PBS before the second HRP labelled antibody was added for 1 hour at room-temperature. Cells were washed 3x with PBS and the substrate 2,2'-azino-bis-(3-ethylbenzo thiazoline)-6-sulfonic acid was added for 10 minutes. The color reaction was stopped with 0.5 N H_2_SO_4_ and the absorbance read at 450 nm (Model 3550; Bio-Rad. Inc., Hercules, CA) [22].

### Involvement Mitogen Activated Protein Kinases (MAPK)

The role of MAPK in proliferation and collagen production was determined in cells pre-incubated with either: 10 μM of the Erk1/2 MAPK inhibitor PD98059, or 10 μM of the p38 MAPK inhibitor SB203580 [9, 22]. The concentration of the chemical inhibitors was selected based on either previous experiments in other cell lines of conditions, or on the recommendation of the distributor (all inhibitors were purchased from Sigma-Aldrich (Buchs, Switzerland).

### Role of IgE receptors

The two IgE receptors and their effect in ECM deposition were studied by pre-incubating cells with 0, 1, or 5 μM of IgE receptor specific siRNAs (Santa Cruz Biotech) for 24 hours. After IgE stimulation, half the concentration of the siRNAs was added every 24 hours as described earlier [9, 22].

### Statistics

The Null-hypothesis was that none of the treatments had any effect on either proliferation, cytokine secretion, or extracellular matrix deposition. To adjust results for small “n” we used the Mann Whiney U-test (unpaired, one-sided).

## Results

The epidemiologic data of the serum donors is provided in “Table 1” and shows no significant differences between the four groups for age and gender. However, the two asthmatic groups presented significant higher serum levels of circulating IL-6 and eotaxin; and the two allergic groups (atopic asthma, atopic non-asthma) had increased IgE serum levels (“Table 1”).

### Serum/disease specific effect on ASMC proliferation

**Fig 1:**
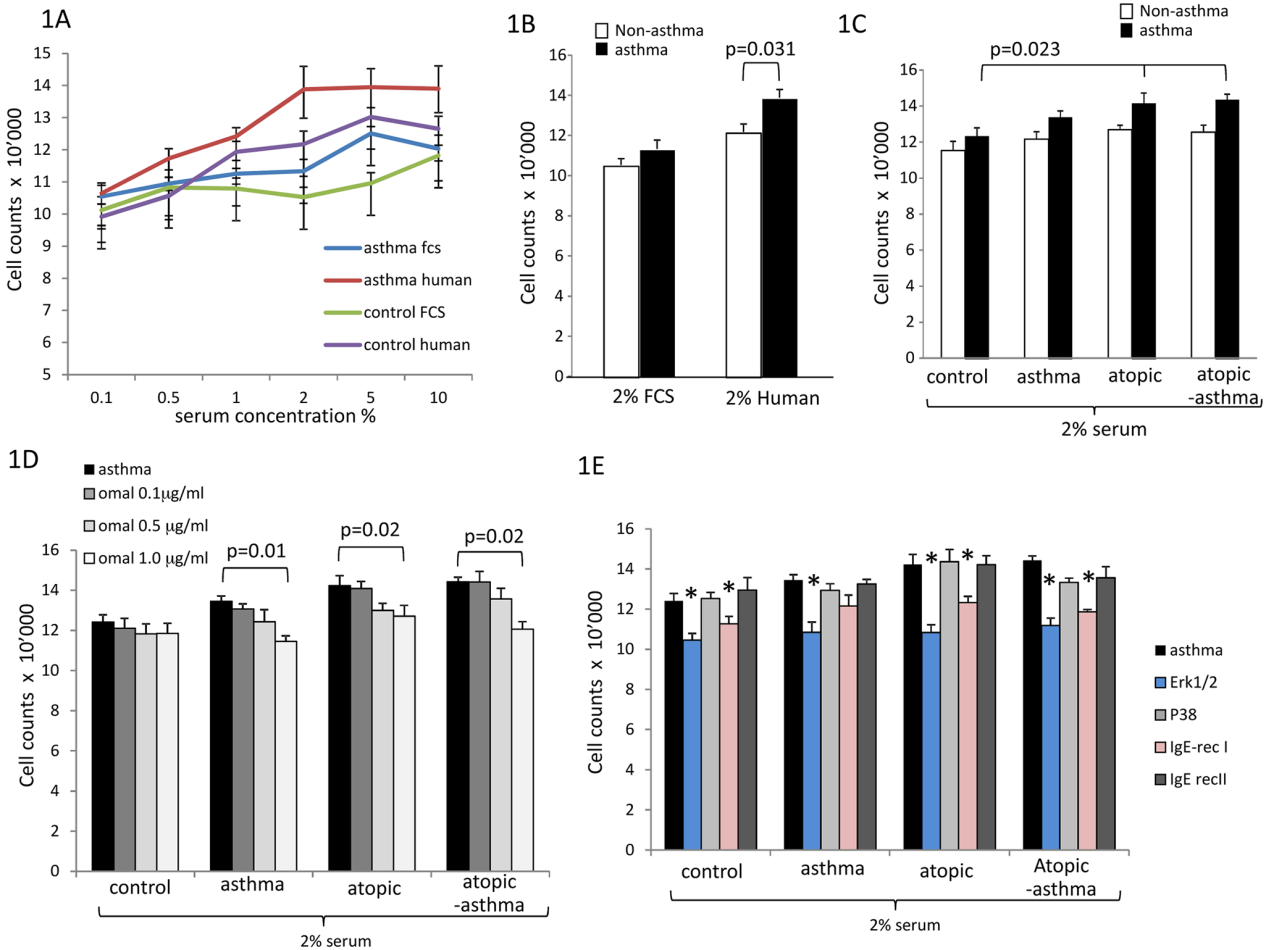
Dose and disease specific effects of serum and IgE on ASMC. (A) Concentration-dependent proliferative effect of human versus fetal calf serum (FCS) on primary ASMC isolated from asthmatic (n = 5) and non-asthmatic (n = 5) patients. (B) Disease specific ASMC proliferation by 2% fetal calf serum or control human serum. (C) Disease and origin specific proliferative effect of four different serum groups (control, atopic-non asthma, asthma, atopic asthma) on asthmatic and non-asthmatic ASMC. Ten sera of each donor group were tested on 5 ASMC of asthma patients and 5 ASMC of non-asthma patients. (D) Inhibitory effect of Omalizumab on serum-induced proliferation on asthmatic ASMC (n = 5). (E) The role of Erk1/2 MAPK and Igε-RI on serum-induced proliferation of asthmatic ASMC. * indicates statistic significant reduction of asthmatic ASMC proliferation compared to serum stimulation (first black bar in each group) with p < 0.05. All presented values present mean±S.E.M.

In ASMC human serum of healthy controls had a much stronger proliferative effect on ASMC and fibroblasts than equal concentrations of fetal calf serum (Fig 1A). Therefore we decided to use 2% human serum as standard concentration for all sub-sequent experiments. Over 5 days 2% serum of healthy controls increased cell numbers by 26% in non-asthmatic and by 35% in asthmatic ASMC which was significant compared to each other (Fig 1B). When grown in 2% serum obtained from atopic non-asthma or atopic-asthma patients ASMC of asthma patients (A-ASMC) proliferated significantly faster than ASMC from all non-asthmatic donors (NA-ASMC) (Fig 1C).

The pro-proliferative effect of atopic donor’s serum on asthmatic ASMC was significantly reduced in the presence of Omalizumab (Fig 1D). Similar data of an inhibitory effect of Omalizumab was obtained in ASMC of non-asthmatic donors (data not shown). Investigating the underlying cellular signaling pathways we observed that atopic serum-induced ASMC proliferation was significantly reduced by inhibition of Erk1/2 MAPK, but not of p38 MAPK (Fig 1E). Down-regulation of Igε-RI by 48 hours siRNA treatment partly reduced ASMC proliferation in all serum types, while inhibition of Igε-RII did not achieve significance (Fig 1E). Addition of mixed asthma relevant allergens did not modify the proliferative effect of atopic serum (data not shown).

### Atopic serum-induced deposition of collagen and fibronectin deposition by ASMC

**Fig 2:**
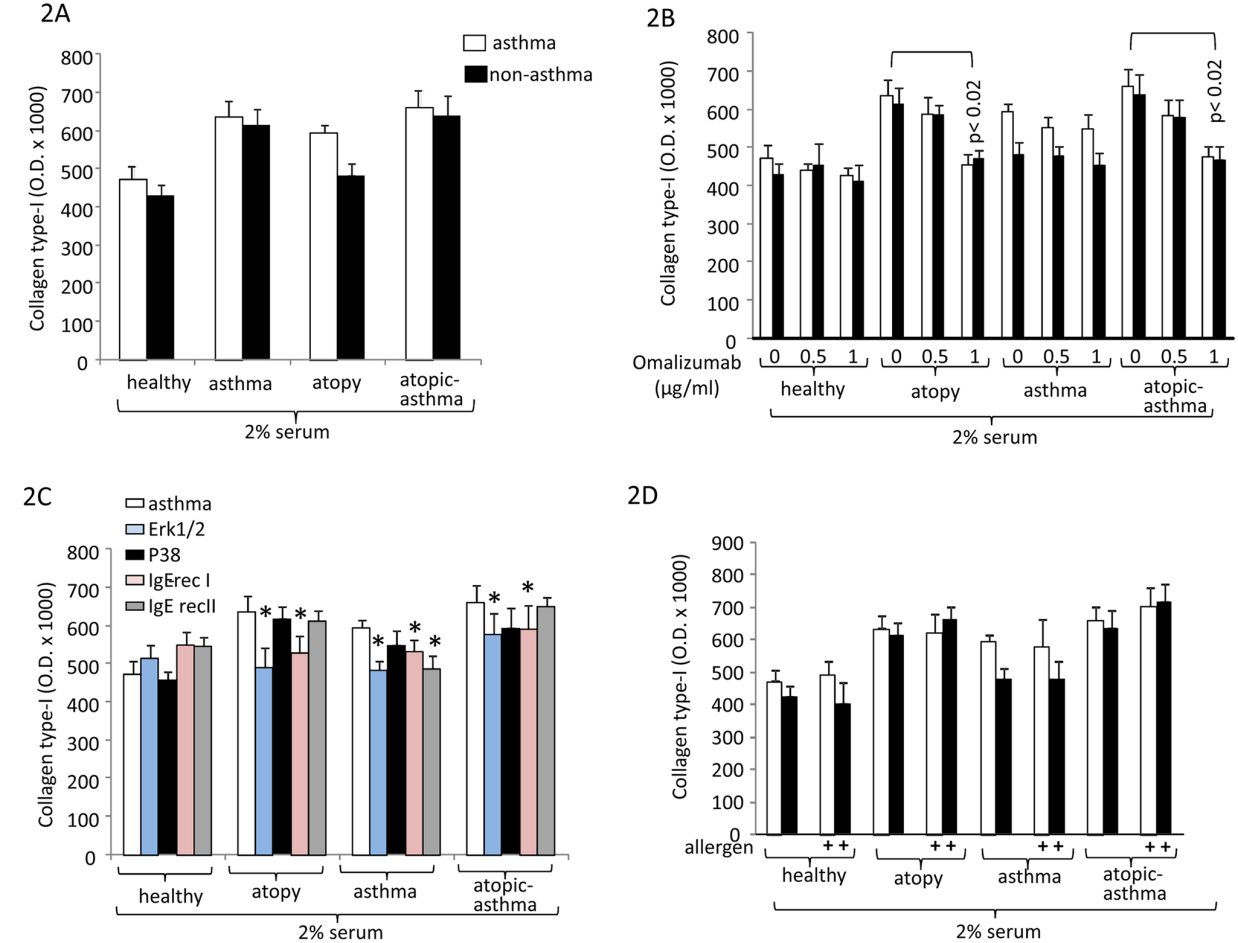
Disease specific increased collagen type-I deposition by ASMC. (A) disease specific collagen type-I deposition stimulating effect of serum (n = 10) at 48 hours. (B) Inhibitory effect of Omalizumab on disease specific serum stimulated collagen type-I deposition by ASMC. (C) Signalling pathways and IgE-receptor mediation of collagen type-I deposition (n = 3). (D) The effect of allergen mix to serum induced collagen type-I deposition (n = 4). All data represent the mean±S.E.M. The optical density measurements of the deposed collagen type I for all experiments are provided as shown in S1 File.

Serum obtained from asthma patients was the strongest inducer for collagen type-I deposition (48 hours), however, with no statistic significant difference comparing serum obtained from patients with atopic or non-atopic asthma (Fig 2A). Atopic serum from non-asthma patients also increased collagen type-I deposition compared to serum of healthy controls, but was less effective than serum from serum of atopic asthma patients (Fig 2A).

Omalizumab reduced the collagen type-I stimulating effect of serum obtained from atopic asthma patients, while this effect did not occur in cells stimulated with serum of non-atopic asthma patients (Fig 2B). Investigating the underlying signaling pathways we observed that collagen type-I was mediated in part by the Igε-RI and Erk1/2 MAPK (Fig 2C). Allergen addition did not further stimulate the serum effect on collagen type-I deposition (Fig 2D).

**Fig 3:**
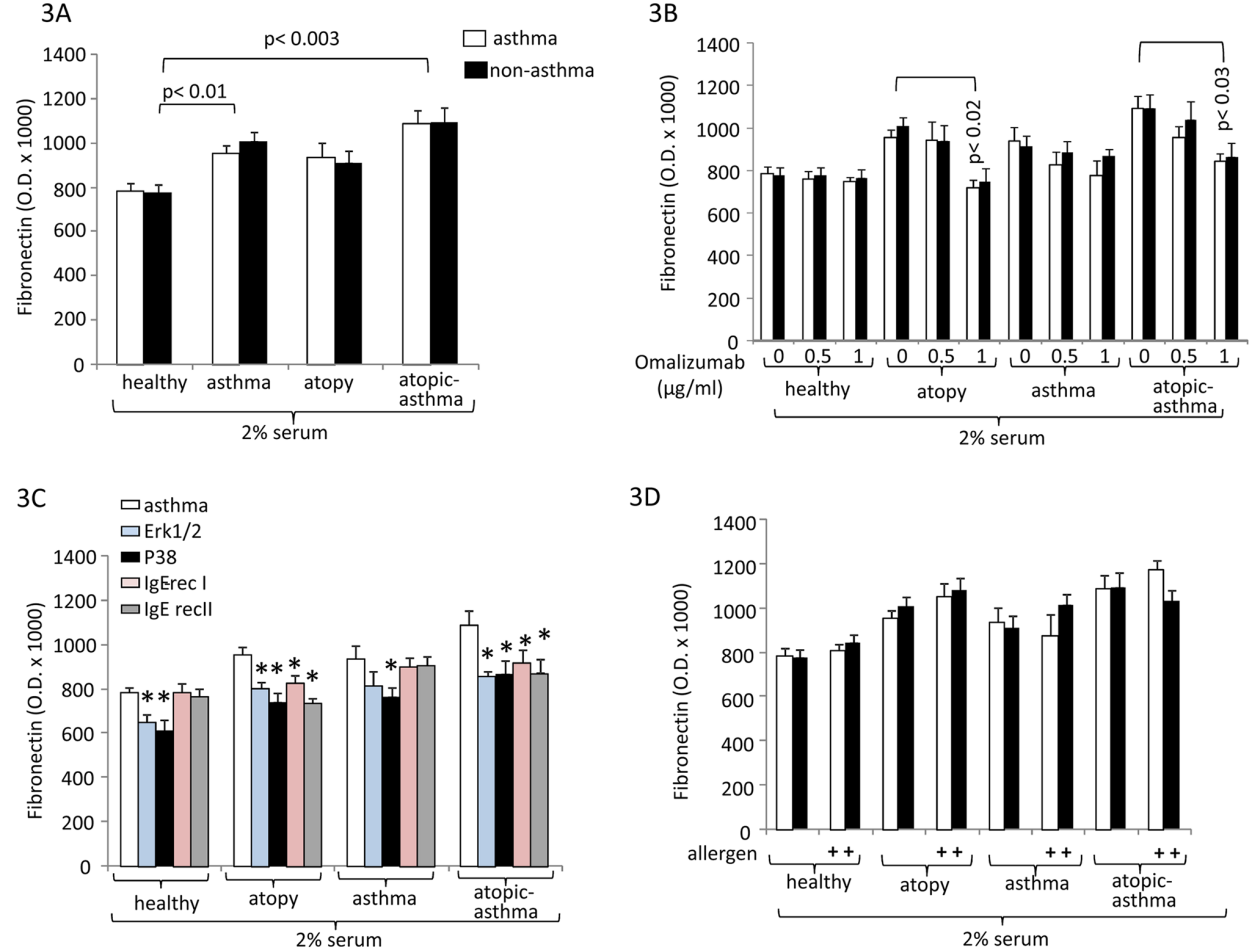
Disease specific deposition of fibronectin by ASMC. (A) disease specific fibronectin deposition stimulating effect of serum (n = 10) at 24 hours. (B) Inhibitory effect of Omalizumab on disease specific serum stimulated fibronectin deposition by ASMC. (C) Signalling pathways and IgE-receptor mediation of fibronectin deposition (n = 3). (D) The effect of allergen mix to serum induced fibronectin deposition (n = 4). All data represent the mean±S.E.M. The optical density measurements of the deposed fibronectin for all experiments are shown in S1 File.

Fibronectin was significantly up-regulated by atopic and asthma serum compared to healthy control serum (Fig 3A), and this effect was dose-dependently reduced by Omalizumab (Fig 3B). However, the inhibitory effect of Omalizumab was only significant at the highest concentration and only in serum of atopic patients and atopic asthma patients, but had no significant effect on serum from asthma patients (Fig 3B). When analyzing the effect of signal transducing MAPK and IgE receptors we observed that fibronectin involved, both Erk1/2 and p38 MAPK and this effect was observed with all sera and indicates that MAPKs are mediating more than the IgE effect (Fig 3C). Regarding the IgE receptors, the down-regulation of both receptors reduced serum-activated fibronectin deposition, but the effect did not reach significance (Fig 3C). Allergens did not increase the stimulating effect of atopic serum significantly (Fig 3D).

## Discussion

**Fig 4:**
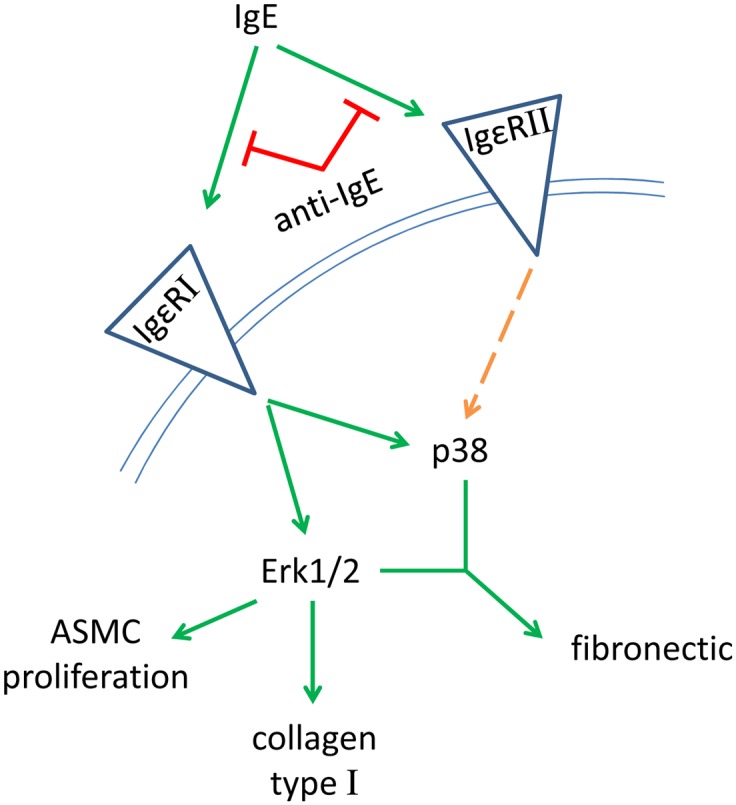
Hypothesized signal pathway for IgE induced airway wall remodeling in human airway smooth muscle cells.

In this study we provide evidence, that circulating IgE is a major contributor to airway wall remodeling in atopic asthma. Serum collected from patients with atopic asthma stimulated mesenchymal cell proliferation and deposition of collagen type-I and fibronectin, mainly by activation of the Igε-RI and Erk1/2 MAPK as summarized in Fig 4. Both, proliferation and matrix deposition were significantly prevented by pre-incubation of the cells with Omalizumab. The findings support the clinically documented beneficial long term effects of Omalizumab on airway structure and function [11, 12].

Omalizumab is a recombinant humanized IgG1 monoclonal antibody which binds and thereby neutralizes circulating free human IgE in the blood [23, 24]. It has been discussed if the concentration of Omalizumab used in in vitro studies is equivalent to that achieved by injection in humans. Assuming that the total blood volume of blood of an average human is 5 liters the most often used amount of injected Omalizumab of 170 mg (MW 145’058.2 kDa) is equivalent to 29 μg/ml or 0.2 μM. In addition, Omalizumab has been shown to inactivated cell membrane bound IgE, at least on B-cells [23]. However, Omalizumab does not bind to IgE which is bound to its high-affinity receptor Igε-RI on mast cells, basophils or dendritic cells [24]. It is intriguing that the principle of anti-IgE antibodies as a counteracting event to allergic response is a naturally occurring event which was only discovered in asthma patients recently [7]. It would therefore be of interest if the effects of the recombinant anti-IgE antibodies are entirely the same as the natural anti-IgE antibodies. In regard to the beneficial effects of Omalizumab on tissue forming mesenchymal cell types we performed some experiments where IgE was added before Omalizumab and no significant reducing effect on either cytokine secretion or extracellular matrix deposition was observed [data not published]. Thus, suggesting that Omalizumab has no effect on IgE which has already bound to Igε-RI.

Here, as well as in earlier studies [9, 21], our results suggest that the high affinity Igε-RI is the major mediator of mesenchymal cell remodeling, both proliferation and matrix deposition, through Erk1/2 MAPK which is in line with other studies [8, 10]. Our observation that added allergens did not further increase the proliferative effect of atopic serum suggests that tissue forming cell do not need a further stimulation by allergens in respond to IgE. In regard to IgE-induced chronic inflammation in atopic asthma it would be of interest if the presence of IgE increases the sensitivity of tissue forming cells to other remodeling relevant proteins such as tumor growth factor-β or tumor necrosis factor-α [25].

Importantly, our data confirms earlier reports of a cell type and disease specific response to atopic serum and IgE, with ASMC obtained from asthma patients responding with faster proliferation compared to ASMC of non-asthma donors while fibroblasts did not show such a disease specific difference [26, 27]. It would be of interest to investigate if this cell type specific effect of IgE [9] and of IgE rich atopic serum has an effect on cell differentiation of fibroblasts into myo-fibroblasts, indicated by the increased expression of α-smooth muscle actin (α-SMA), as it had been suggested in different conditions [26].

Cell differentiation and function depends on the local composition of the extracellular matrix [28]. Here, the ratio of the different components to each other is more important for the effect on the embedded cells, than the expression itself [29]. The observed increase of collagen type-I and fibronectin by IgE rich atopic serum or by IgE alone [19], suggests that both ASMC and fibroblasts create a pro-inflammatory condition in the airways of asthma patients. Similar to our earlier report on IgE mediated increased deposition of collagen type-I, IgE rich serum achieved its effect through the Igε-RI and Erk1/2 MAPK (Fig 4). In contrast, the up-regulation of fibronectin by IgE rich serum or IgE [19] was independent of this signaling pathway. However, there are still open questions regarding the mechanism(s) how IgE and its neutralizing antibodies affect airway wall remodeling and function. Why do tissue forming cells express IgE receptors and why do they respond to them in the absence of allergens? It is unknown by which mechanisms IgE gets access to sub-epithelial mesenchymal cells *in vivo*. Does circulating IgE have an organ specific effect on tissue forming cells in the asthmatic airways only or does such an action also occur in other organs. Does the presence of IgE “prepare” tissue forming cells for further inflammatory signals and lower their threshold for such signal mediators? Answering this question may also help to understand the beneficial effects that have been reported for Omalizumab in clinical studies.

In summary, the question if Omalizumab can reduced airway wall remodeling in atopic asthma, raised by Rabe in 2011 [29], can be answered positive. Clinical [30, 31] and experimental studies prove that neutralizing IgE has a reducing effect on airway wall remodeling which importantly cannot be achieved by other therapeutic strategies in asthma.

## Supporting Information

S1 File OD for collagen type I deposition after 48 hours serum treatment. This was determined by the in house developed ELISA and described earlier [9,18,21,22]. The values represent the mean of dual measurements for each data point. Since the ELISA detects the ECM compounds as they are deposed by the cells there are no standard curves available.
(DOCX)
