# Supplementary material for: Serum IgE Induced Airway Smooth Muscle Cell Remodeling Is Independent of Allergens and Is Prevented by Omalizumab
Source: PLoS One. 2015 Sep 2;10(9):e0136549. doi: 10.1371/journal.pone.0136549 (PMC4557956; doi:10.1371/journal.pone.0136549)

OD for collagen type I deposition after 48 hours serum treatment


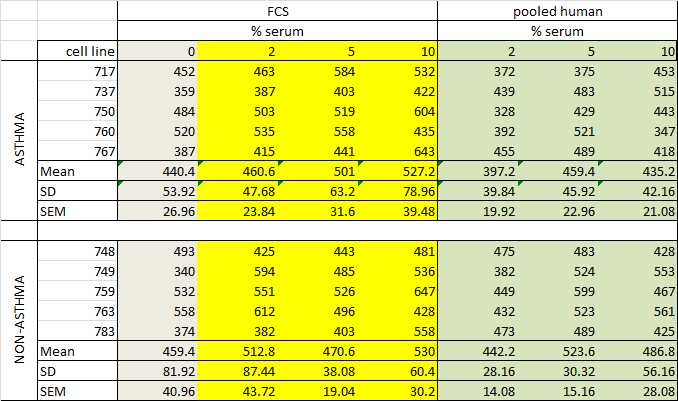


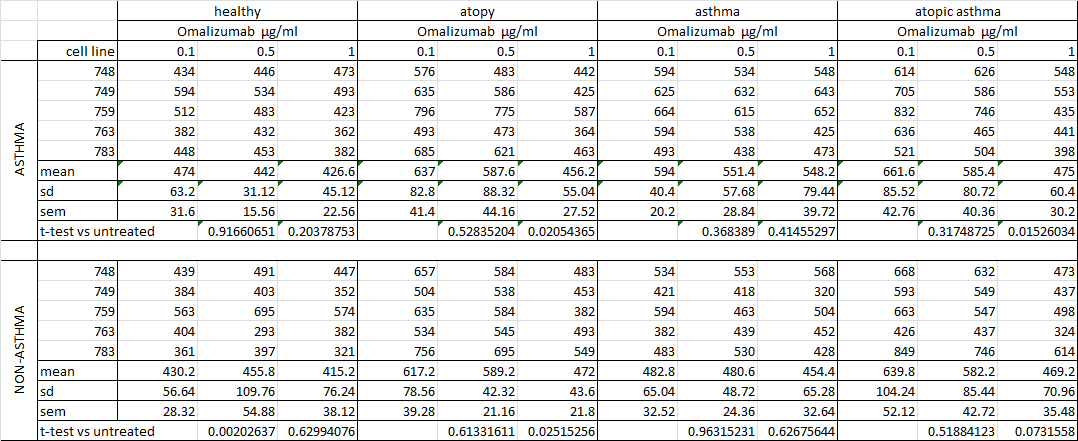


Optical Density for fibronectin deposition after 24 hours serum treatment


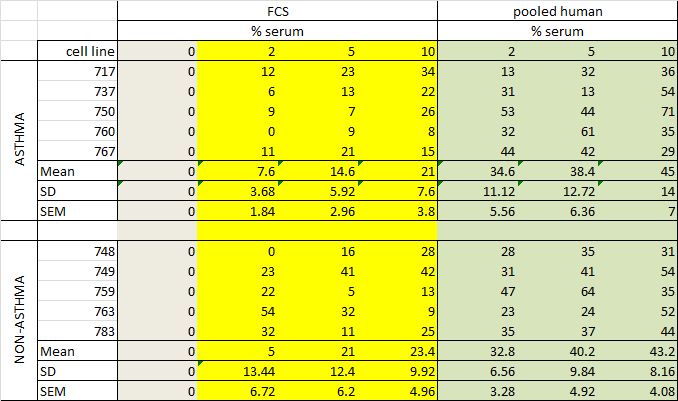


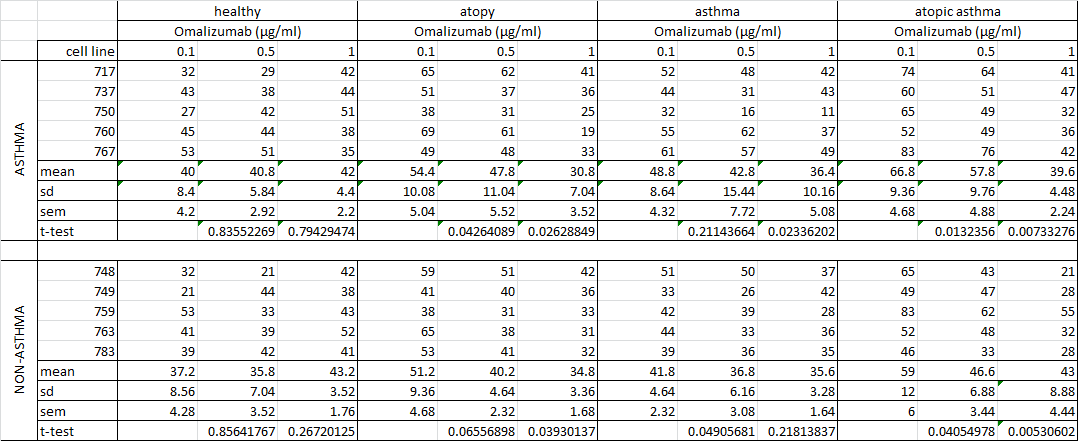


Optical Density for collagen type I deposition after 48 hours serum treatment


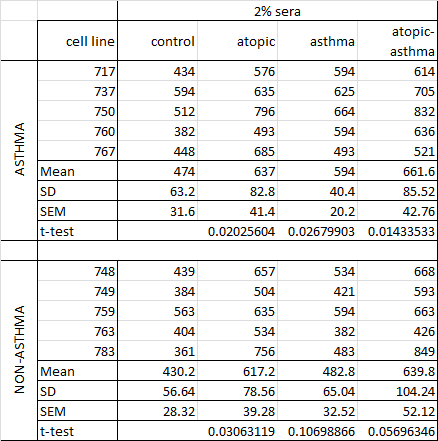


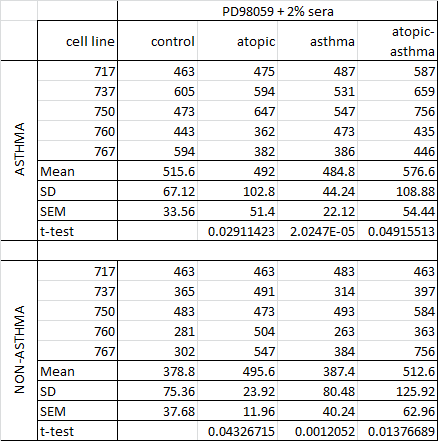


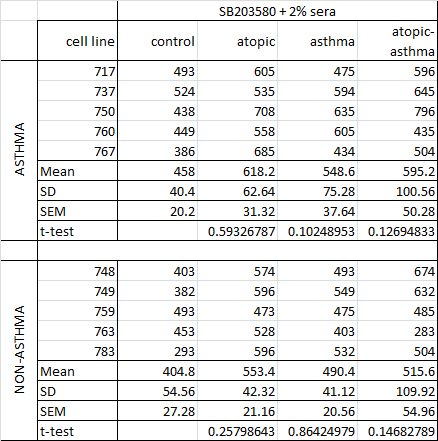


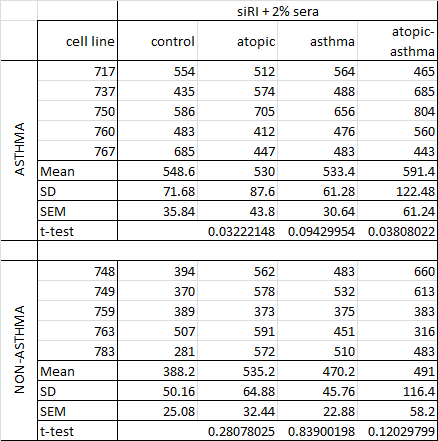


|  |  | siRII + 2% sera | | | |
| --- | --- | --- | --- | --- | --- |
|  | cell line | control | atopic | asthma | atopic-asthma |
| ASTHMA | 717 | 535 | 664 | 434 | 645 |
|  | 737 | 534 | 586 | 532 | 705 |
|  | 750 | 574 | 687 | 605 | 697 |
|  | 760 | 437 | 495 | 401 | 538 |
|  | 767 | 645 | 621 | 463 | 663 |
|  | Mean | 545 | 610.6 | 487 | 649.6 |
|  | SD | 51.6 | 56.08 | 65.2 | 46.48 |
|  | SEM | 25.8 | 28.04 | 32.6 | 23.24 |
|  | t-test |  | 0.47652983 | 0.02477329 | 0.81897897 |
|  |  | | | | |
| NON-ASTHMA | 748 | 483 | 688 | 591 | 808 |
|  | 749 | 408 | 637 | 587 | 676 |
|  | 759 | 439 | 421 | 423 | 432 |
|  | 763 | 496 | 579 | 441 | 309 |
|  | 783 | 345 | 703 | 627 | 594 |
|  | Mean | 434.2 | 605.6 | 533.8 | 563.8 |
|  | SD | 46.16 | 84.48 | 81.44 | 154.64 |
|  | SEM | 23.08 | 42.24 | 40.72 | 77.32 |
|  | t-test |  | 0.85265949 | 0.44102806 | 0.39870646 |

Optical Density for fibronectin deposition after 24 hours serum treatment


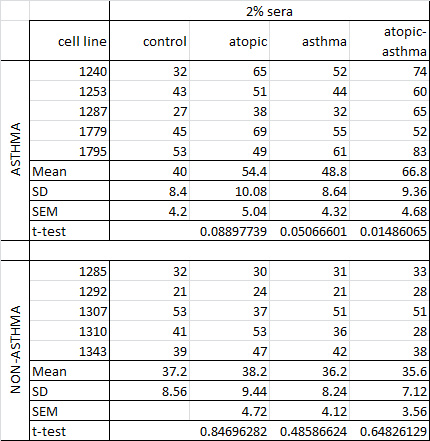


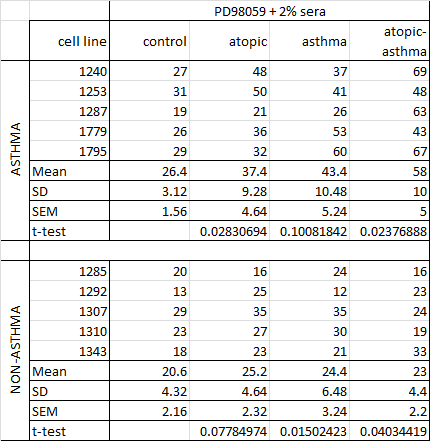


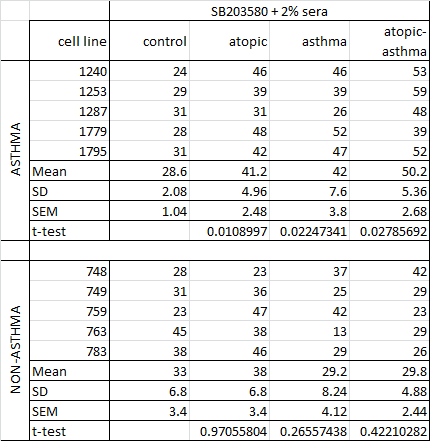


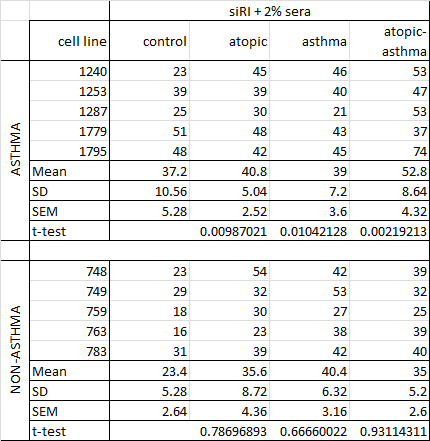


|  |  | siRII + 2% sera | | | |
| --- | --- | --- | --- | --- | --- |
|  | cell line | control | atopic | asthma | atopic-asthma |
| ASTHMA | 1240 | 43 | 42 | 45 | 72 |
|  | 1253 | 56 | 63 | 52 | 63 |
|  | 1287 | 39 | 44 | 64 | 71 |
|  | 1779 | 42 | 55 | 49 | 63 |
|  | 1795 | 19 | 51 | 49 | 58 |
|  | Mean | 39.8 | 51 | 51.8 | 65.4 |
|  | SD | 8.64 | 6.4 | 4.96 | 4.88 |
|  | SEM | 4.32 | 3.2 | 2.48 | 2.44 |
|  | t-test |  | 0.62977204 | 0.72588687 | 0.83414349 |
|  |  | | | | |
| NON-ASTHMA | 748 | 31 | 56 | 44 | 43 |
|  | 749 | 40 | 36 | 36 | 40 |
|  | 759 | 28 | 29 | 35 | 53 |
|  | 763 | 51 | 43 | 42 | 47 |
|  | 783 | 21 | 35 | 46 | 54 |
|  | Mean | 34.2 | 39.8 | 40.6 | 47.4 |
|  | SD | 9.04 | 7.76 | 4.08 | 4.88 |
|  | SEM | 4.52 | 3.88 | 2.04 | 2.44 |
|  | t-test |  | 0.8408173 | 0.46859555 | 0.0153269 |

Optical Density for collagen type I deposition after 48 hours exposure to allergens


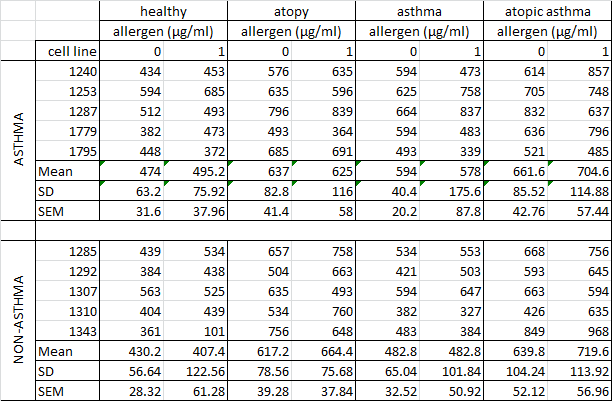


Optical Density for fibronectin deposition after 24 hours exposure to allergens


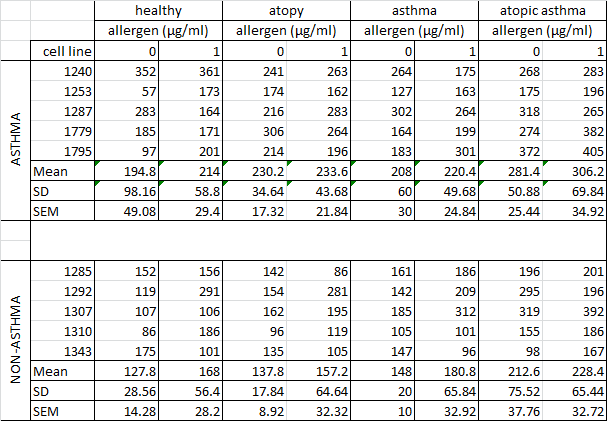

Supplement: S1 File — This was determined by the in house developed ELISA and described earlier [9,18,21,22]. The values represent the mean of dual measurements for each data point. Since the ELISA detects the ECM compounds as they are deposed by the cells there are no standard curves available. (DOCX) [file pone.0136549.s001.docx]
